# Addressing the challenges of including older people with osteoarthritis and multi-morbidity in digitally enabled trials: a mixed methods Study Within a Project (TIPTOE SWAP)

**DOI:** 10.1186/s13063-026-09907-w

**Published:** 2026-07-27

**Authors:** Victoria Shepherd, Hayley Prout, Kate Button, Lucy Brookes-Howells, Elizabeth Randell, Rachel Lowe, Sarah Nash, Rachel Deere, Monica Busse

**Affiliations:** 1 Centre for Trials Research, Cardiff University, 6th floor Neuadd Meirionnydd, Heath Park, Cardiff, CF14 4YS UK; 2 School of Healthcare Sciences, Cardiff University, Cardiff, UK; 3 Division of Digital Health and Applied Technology Assessment, Faculty of Nursing, Midwifery & Palliative Care, King’s College London, London, UK

**Keywords:** Osteoarthritis, Multiple long-term conditions, Multimorbidity, Older adults, Inclusivity, Study within a project (SWAP)

## Abstract

**Introduction:**

Older adults, particularly those with multiple long-term conditions (MLTCs) and other intersectional characteristics, are under-represented in clinical trials. This includes digitally enabled trials as older people may be less likely to engage with digital methods.

We embedded a ‘Study Within a Project’ (SWAP) in TIPTOE, a UK randomised controlled trial evaluating a self-management support intervention for older adults with knee and/or hip osteoarthritis and MLTCs. Osteoarthritis is a musculoskeletal condition that is more common with age. The aim of the SWAP was to understand the barriers to including older people with MLTCs, rapidly develop strategies to support inclusion, and provide methodological guidance for future trials including those that are digitally enabled and/or decentralised.

**Methods:**

The mixed-methods SWAP spanned three trial phases: (1) set-up, process mapping to establish recruitment pathways and characteristics of local clinical populations; (2) pilot, interviews with recruiting staff to explore attitudes, skills and confidence when recruiting participants from under-served groups; (3) transition to main trial, interviews with participants to explore their experiences of taking part. Rapid analysis enabled changes to be made to trial processes during the early stages of the trial.

**Results:**

Process mapping identified a diversity of populations and accessibility considerations (e.g. language, unfamiliarity with digital technology). Interviews with recruiting staff (*n* = 12) identified potential recruitment challenges including concerns that older people may be less inclined to participate due to lower digital literacy or lack of understanding about the trial. Interviews with participants (*n* = 25) found even those with digital access preferred human support and were concerned about being ‘scammed’ when receiving trial communications. Strategies were introduced such as lowering the eligibility age and creating tailored materials and messaging. Recommendations for future trials include ensuring person-centred support for participants, which has resourcing implications.

**Conclusion:**

Embedding a SWAP in TIPTOE enabled a greater understanding about recruiting older people who experience intersectional barriers to inclusion. Approaches to support recruitment included providing telephone support for those less confident navigating digital consent and data collection processes and the creation of guidance for sites. The findings could support future trials to better include older people with MLTCs and other under-served groups.

**Trial registration:**

ISRCTN 16024745. Registered on October 16, 2023.

**Supplementary Information:**

The online version contains supplementary material available at https://doi.org/10.1186/s13063-026-09907-w.

## Introduction

Osteoarthritis (OA) is a musculoskeletal condition which, whilst it can affect people of all ages, is more common with increasing age [1]. However, older adults are under-represented in OA trials, limiting the applicability of current evidence to this growing population [2]. As a consequence, little is known about the short-term and long-term benefits of non-pharmacological and pharmacological treatments for OA in older people [3]. The need to urgently improve the inclusion of older people in research was recently highlighted in a joint call from leading UK charities and research institutes who warn that the development of new treatments is being undermined by this under-representation, particularly when compared with the burden of disease, which undermines the usefulness and accuracy of study findings [4].

Older people are frequently excluded from clinical trials due to the presence of multiple long-term conditions (MLTCs) and polypharmacy, difficulties accessing research opportunities, concerns around cognitive impairment and the ability to provide consent, and gatekeeping by healthcare professionals and family members [5]. In addition, older people are less likely to actively seek participation in clinical trials, although they often report high levels of willingness to participate [5]. Alongside older people and those with MLTCs (alternatively termed multimorbidity), a number of other groups have been identified as being under-served by research, including people from ethnic minority groups, people living in remote areas, socio-economically disadvantaged groups, and those with cognitive impairment which affects their ability to provide their own consent [6–8]. For many of these groups, these research inequalities contribute to the widening health inequalities they already experience. Initiatives such as NIHR INCLUDE have called for improvements in the design and delivery of trials to ensure that more people from under-served groups have the opportunity to participate in research and to ensure the delivery of better healthcare for these groups [6]. More recently, there has been a growing focus on intersectionality, in which multiple social categories, such as age, disability, and socio-economic status interact to form social experiences that are distinct from the sum of their parts, and exclusion from trials [9]. An overlap has been identified between people with intersecting characteristics who are under-served by research and those who are less likely to engage with digital research methods—such as informed consent (‘e-consent’), data collection, and research communications [10]. This has led to calls to develop effective approaches for optimising diverse engagement with digital methods [10].

Clinical trials are increasingly being delivered outside of hospitals or research centres as this is thought to reduce some of the barriers to participation in trials, including for those experiencing ill health and reduced mobility, or those who encounter challenges with transport or with external commitments such as caring responsibilities [11]. In these decentralised trials (DCTs), some or all trial-related activities occur at locations other than traditional clinical trial sites, for example, in participants’ homes or local clinics [12]. However, whilst remote research delivery may be beneficial for some in terms of accessibility and equity [13], caution must be taken not to exacerbate inequalities through digital exclusion, which may particularly be the case for older people who may not have access to suitable equipment or the internet, or may be hesitant due to a lack confidence or privacy concerns [14]. This has led to calls for greater consideration about who may be excluded from remotely conducted studies including DCTs [14].

TIPTOE is a randomised controlled trial to evaluate the clinical and cost effectiveness of a co-designed personalised self-management support intervention for older adults with knee and/or hip OA and MLTCs in the UK, compared with usual NHS care [15, 16, 17]. To date, the development and evaluation of many self-management interventions have not specifically focused on frailer older people nor focused on the challenges of inclusion in trials [18]. In TIPTOE, participants are recruited through several methods including self-referral via the trial website, and they are able to provide informed consent, complete questionnaires, and receive the intervention remotely if they wish. In order to understand and address the barriers to including older people with MLTCs in TIPTOE, we embedded a ‘Study Within a Project’ (SWAP) in the TIPTOE study.

The aim of the SWAP was to identify the barriers to the inclusion of people living with OA and MLTCs (including frailty) in TIPTOE, with a focus on the intersection between MLTCs and other under-served groups (e.g. ‘oldest old’, ethnic minorities, digitally excluded). The intention was to rapidly address the barriers through the development of tailored approaches to support the recruitment and follow-up of these groups. We also wanted to provide methodological guidance for future trials of complex interventions to better include older people with MLTCs and other under-served groups, thus contributing to the growing body of evidence around recruitment of these populations more broadly.

## Methods

A full description of the study design and analysis plan for TIPTOE and for the process evaluation and economic evaluation can be found in the protocol which has previously been published [15]. Briefly, participants (older people with knee and/or hip joint pain from their OA affected joint and at least one other long-term health condition) were recruited from Wales and England through several methods including self-referral via the TIPTOE study website (https://www.tiptoe.org.uk/), being approached during routine NHS clinic attendance (e.g. at musculo-skeletal physiotherapy outpatient clinic), or waiting list screening and invitation letters (e.g. waiting lists for musculo-skeletal physiotherapy or for joint replacement surgery), through primary care, and via third sector and voluntary community organisations. 

Participants were eligible for TIPTOE if they were able to provide informed consent and as follows:

1. Were age 70 + years (protocol later amended to 65 + years—see Results section)
2. Were living in the community independently, with carer support or in assisted living
3. Had self-reported knee and/or hip pain in the OA-affected joint, which is impacting upon daily living
4. Had knee and/or hip OA and at least one other co-morbidity identified using the self-administered comorbidity questionnaire or OA and mild to moderate frailty on the CHSA Clinical Frailty Scale (for clinician referral routes only).

They were excluded if they are as follows:

1. Had joint pain associated with a malignant condition
2. Have had knee and/or hip surgery or joint replacement surgery in the affected joint in the last 12 months
3. Were living in a care home (residential or nursing care)
4. Were unable to engage with the intervention, even when provided with tailored support (based on clinician assessment)

Following informed consent, participants were randomised 1:1 to receive either usual NHS care or usual NHS care plus the Living Well support intervention, which included being offered up to six one-to-one coaching sessions with a TIPTOE-trained healthcare practitioner and a co-designed book. Participants were encouraged to nominate a support person to assist them throughout the study. All participants were asked to complete a series of self-reported outcome measures at baseline and 6- and 12-month follow-up. The primary outcome was symptoms and quality of life as assessed by the Musculoskeletal Health Questionnaire (MSK-HQ). Routine data was used to evaluate health resource use, and wearable activity monitors (ActivPal) were used to measure activity in a subsample of participants. A mixed methods process evaluation was also conducted to explore trial processes, intervention mechanisms, and context, to inform further implementation. This included interviewing a sub-set of healthcare practitioners (HPs) and participants during the pilot and main trial phases. Extensive patient and public involvement and engagement (PPIE) input was embedded throughout the project, following the UK Standards for Public Involvement [19], from the co-design of the TIPTOE intervention through to the development of the trial protocol and patient-facing documents and materials.

Informed by guidance around the inclusion of older people [3, 14, 20] and under-served groups more broadly, extensive efforts were made to ensure inclusivity for this study population [6]. The TIPTOE intervention was systematically developed using a six-step Intervention Mapping approach, supplemented with Accelerated Experience-Based Co-Design methodology. It incorporated stakeholder feedback at every stage, from initial problem definition through narrative interviews and co-design group meetings to final refinement of the ‘Living Well with Joint Pain’ book and training materials. Deliberate efforts were made to include the lived experiences of diverse participants and facilitate meaningful participation of individuals of different ages, genders, ethnic backgrounds, and geographic regions across Wales and England. To maximise accessibility across different healthcare contexts and populations, the intervention was intended to be delivered by various healthcare professionals (physiotherapists, nurses, occupational therapists) through multiple formats (online, in-person clinic, or home visits), directly responding to stakeholder input about practical implementation needs.

Participants were able to be recruited, complete questionnaires, and receive the intervention via a range of routes including online, by phone, or in person—which potentially included their own home where local arrangements could support this—or a combination. During the development of the trial, the proposed design was reviewed by the TIPTOE team using the NIHR INCLUDE Impaired Capacity to Consent Framework [21] to ensure it was as accessible as possible for people with cognitive impairment. This resulted in amendments relating to accessibility of the participant information materials, the development of a summary information sheet to help with layering of information to support comprehension and decision-making, and a central email account and telephone number which was closely monitored by the central TIPTOE team (which included experienced research nurses), to provide additional support for participants where needed.

Building on this initial development work, the SWAP used a mixed-methods approach to explore the methodological and systemic barriers to including older people with MLTCs in the TIPTOE study through three phases:

1. Pre-trial: during the set-up phase of the trial, the aim was to establish the characteristics of the clinical population(s) through working collaboratively with sites to map recruitment pathways to local care pathways with a focus on the inclusion of groups who have been identified as being under-served by research [6].
2. Pilot phase: during the pilot phase of the trial, the aim was to explore trial recruiters’ attitudes, skills and confidence when recruiting participants from populations that are under-served, through interviews with members of research teams at recruiting sites.
3. Transition from pilot to main trial: during the transition phase of the trial, the aim was to explore participants’ experiences of taking part in the trial through interviews with participants with a particular focus on those from under-served groups.

### Recruitment and data collection

#### Phase 1: mapping of recruitment pathways at sites

Sites were originally intended to total eight sites across four UK regions (Wales, Southern England, Southeast and London, and Southwest England). Site selection criteria were based upon the identification of regions which traditionally have low recruitment to MSK research and have high numbers of people within their population from under-served groups in this context, e.g. people with MLTCs (with and without frailty), including those with cognitive impairment, ‘oldest old’ (80 + years), and other intersectional factors such as people from ethnic minority groups, people living in remote areas, and those who experience socio-economic disadvantage. During the pre-trial and pilot phase, the number and types of sites were increased from those originally planned to address the lower than anticipated recruitment rate. This meant that by March 2025 there were 18 sites open to recruitment across a wider geographical area with the addition of four regions in England (Midlands, North, Northwest, and South). This included GP practices in Southwest England and a further cluster of third sector community organisations who were part of a programme to provide additional support for people aged 60 + that was funded by an NHS Trust.

During a second phase of recruiting sites and widening the opportunities for different communities to participate, recruitment routes were also expanded to include ‘Be Part of Research’ (https://bepartofresearch.nihr.ac.uk/) which is an online registry where members of the public can be matched to suitable studies depending on which health conditions they are interested in, and an additional 20 GP practices in Wales acting as Participant Identification Centres (PICs) which are organisations that identify potential research participants for a research study (e.g. through search of patient databases) and have no other involvement in the research.

Recruiting sites were contacted by a member of the TIPTOE central team when they were in the process of being set up (e.g. when a site initiation visit (SIV) was being arranged) and invited to take part in a discussion to explore how to improve inclusion when recruiting participants. For most sites this included arranging an online meeting to discuss their recruitment plans, map recruitment pathways to local care pathways with a focus on any barriers to the inclusion of groups who are considered under-served in this context, and what might be helpful in terms of widening access to the trial. The meeting would last approx. an hour and could include the local Principal Investigator (PI) and any members of the site team they thought it would be useful to include.

Site teams were sent recruitment pathways mapping questionnaire to complete ahead of the online meeting (see Supplementary file). This contained sections for site contact details followed by a series of questions about the site:

- Local clinical population (including those who might be considered to have characteristics of under-served groups).
- Access to eligible participants, including those from under-served groups, and whether there are any barriers that may affect recruitment and participants’ ability to access and participate in TIPTOE at their site.
- Enrolment/registration and consent processes, and whether there are any strategies that could support recruitment to TIPTOE at their site, including those from under-served groups.
- Data collection processes and whether there are any strategies that could support participation in TIPTOE at their site, including those from under-served groups.
- Intervention delivery, and whether there are any strategies that could support participation in and access to the TIPTOE intervention at their site, including those from under-served groups.

The meeting started with a discussion about why there is a focus on inclusivity in TIPTOE, followed by the questions listed in the Word document and their responses. The Word document was then updated by the site and/or TIPTOE team.

An alternative process was developed for GP practices as they had limited capacity to arrange a separate meeting, with two slides being added into the standard SIV slide set to cover the reason for focusing on inclusivity in TIPTOE and four key questions to facilitate discussion:

1. Clinical population—how would you broadly describe the local clinical population, particularly in terms of characteristics of under-served groups?
2. Recruitment pathways—how do you plan to identify participants for TIPTOE (e.g. clinics, searches of patient lists).
3. How might particular aspects of the trial impact on the ability to identify and include under-served groups at this site, for example:
  Registration and consent process
  Data collection
  Intervention delivery
  Other aspects of the trial
4. Are there any particular requirements that should be considered when setting up the study at your site?

#### Phase 2: exploring research teams’ attitudes, skills and confidence in recruiting under-served groups

During the pilot phase, a series of one-to-one interviews were conducted to explore the attitudes, skills, and confidence of site staff who were recruiting participants. To reduce the burden for interviewees, these could be combined with process evaluation interviews when being conducted at the same timepoint if the site staff had a dual role—meaning they were also the healthcare professionals who were delivering the TIPTOE intervention. We aimed to interview approximately 10 recruiters.

After site initiation, recruiting staff were identified through the study database and discussions with the TIPTOE central team and purposefully sampled for maximum variation across regions and patients recruited through different pathways (e.g. musculoskeletal services, community service). Staff at organisations that were involved in alternative recruitment routes which had limited or no direct contact with potential participants (i.e. Be Part of Research registry) were not invited to take part in an interview.

Recruiting staff were contacted by a qualitative researcher (HP) to confirm their role in the TIPTOE study, provided with a Participant Information Sheet and consent form, and invited to take part in a telephone/video interview. Consent was sought verbally at the start of the interview and audio recorded. Interviews were semi-structured and informed by the topic guide developed for the study (see Appendix), and all were conducted by one qualitative researcher (HP). Interviews were audio recorded and transcribed verbatim using a professional transcription service and de-identified.

#### Phase 3: participants’ experiences of taking part in the trial

During the transition between the pilot and main trial phase, a series of one-to-one interviews were conducted with participants (with the support of their carer/nominated supporter where appropriate) to explore their experiences of participating in the trial (e.g. recruitment, consent, data collection). We purposefully sampled for maximum variation across regions and sites where participants were recruited through different pathways (e.g. musculoskeletal services, community service) with a focus on diversity and under-served groups such as those population characteristics noted in the pre-trial mapping phase. This could include disability including impaired cognitive function that may have affected their ability to engage (or remain engaged) with the intervention, age, ethnicity, socio-economic disadvantage, and other characteristics. In order to reduce the burden for interviewees, these could be combined with process evaluation interviews when being conducted at the same timepoint, which were generally timed to be around baseline or early in their participation in the trial or 6 months post-randomisation. We aimed to interview approximately 15 participants (10 from intervention arm and 5 from usual care arm).

At time of consent to trial participation, participants could tick a box on the consent form to indicate that they would like to be considered to take part in interviews and agree to be contacted to participate in interviews with a TIPTOE trial team member. Participants were contacted by a qualitative researcher (HP) and provided with an interview-specific Participant Information Sheet and consent form and invited to take part in a telephone or video interview. Consent was sought verbally at the start of the interview and audio recorded. Interviews were semi-structured and informed by the topic guide developed for the study (see Appendix), and all were conducted by one qualitative researcher (HP). Interviews were audio recorded and transcribed verbatim using a professional transcription service and de-identified.

### Data analysis

In phase 1, we used a process mapping approach [22, 23] to review the recruitment pathways mapping questionnaires completed by/with the study sites and map the different recruitment pathways into the study. Responses to the questions were reported narratively, grouped according to the area of the trial the question related to (local clinical population, access to eligible participants, enrolment/registration and consent processes, data collection processes, and intervention delivery).

For phases 2 and 3, we adopted the principles of rapid qualitative analysis [24] for the SWAP data, alongside the thematic analysis approach [25] that will subsequently be used for the PE data. Rapid methodologies have been found to be a useful approach when aiming to increase the participation of under-served groups [26].

A ‘RREAL sheet’ was developed for TIPTOE, which is a structured tool to help synthesise data and report emerging findings while further data collection is still ongoing [26]. As a flexible tool, it can be used to discuss emerging findings in real-time with stakeholders and summarised in a one-page table [26]. The categories used in the RREAL Sheet may be used as an initial coding framework for future thematic analyses to be conducted as part of the process evaluation [26]. Analysis was conducted by a team of two qualitative researchers (HP, VS) with input from a third (LBH).

We used a systems-based approach to triangulate the findings from phases 1–3 to identify barriers to the inclusion of people from under-served populations at either an individual, recruiter/site, or trial level. The findings were used during the early stages of the trial to help refine the TIPTOE recruitment processes to ensure they were as inclusive of these under-served groups as possible. They were also used to develop facilitative strategies for rapid implementation during the main trial such as additional training and tailored resources to support research teams to recruit under-served groups. Alongside the process evaluation, the SWAP findings were also intended to inform the implementation of the TIPTOE intervention in the future (beyond the trial) should it be found to be effective, and to provide evidence-based strategies to support inclusivity in future complex intervention trials.

## Results

### Phase 1: mapping of recruitment pathways

**Fig. 1:**
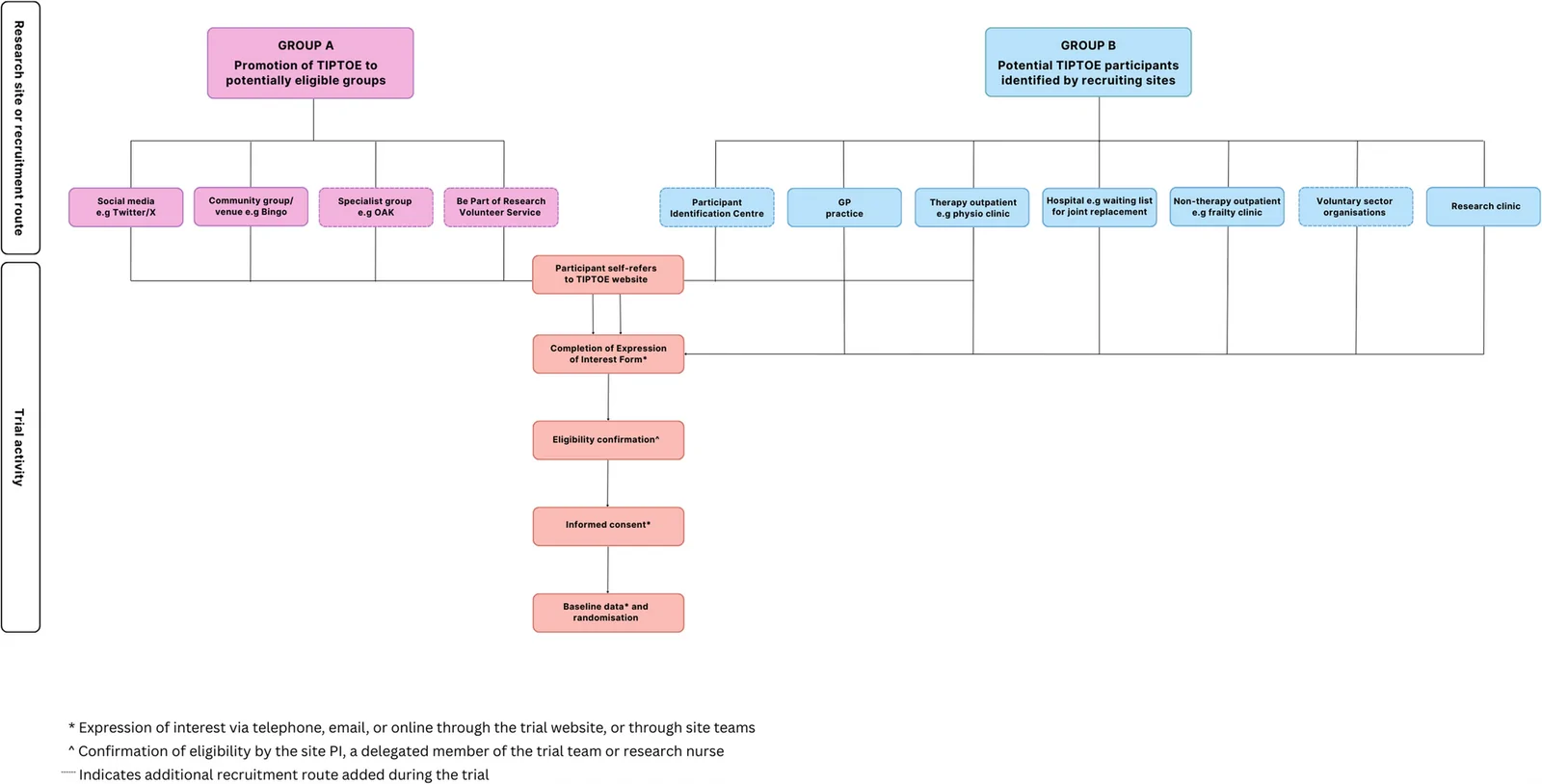
Map of TIPTOE recruitment pathways

In response to slower recruitment rates than anticipated during the pilot phase of the trial, plans for the number and types of research sites that would be involved in recruiting participants for TIPTOE evolved between the start of the trial and the intended end date of the SWAP. This led to a broader range of settings for recruitment activity, such as third sector community organisations and an online research registry (Be Part of Research) and also meant differences in where trial activities such as identification, recruitment, eligibility confirmation, and delivery of the intervention were taking place, and who was involved. Figure 1 shows how research sites or recruitment routes into the trial can be split into those where TIPTOE was promoted to broad groups of people who were likely to be potentially eligible (e.g. via social media, community venues) (Group A) and where potentially eligible individuals were identified and provided with information about the trial (e.g. GP practices, PICs, physio clinics) (Group B). Depending on the setting and the potential participant involved, they would either self-refer and navigate the expression of interest process via the website or be specifically directed by staff to complete the expression of interest. Some Group B sites used both routes (e.g. a GP practice might display a poster in the general waiting room and also screen patient lists to identify eligible patients). In all cases, they could express an interest online or receive support by telephone from a member of the site team (where possible) or the central TIPTOE team.

Mapping questionnaires were only completed by/with the Group B sites as the questions were not designed to apply to generic recruitment routes such as those in Group A (e.g. social media or research registries). A total of 13 questionnaires were completed. The diverse range of responses and the differing issues around inclusivity raised by the sites are shown through the verbatim examples shown in Table 1 (inclusivity aspects highlighted in italics).

**Table 1:** Examples of verbatim responses to the site mapping questionnaire

Local clinical population
This is a very bilingual area with a lot of first ***language*** Welsh people especially in the West of the region which is very ***rural***. All ***age groups*** but as it is a retirement area it is quite an elderly population. Predominantly ***white population***
Coastal area. Historically a retirement area, for example 25% of the population are ***over 75***. Predominantly a middle/working class and well-educated population. Mainly ***White British***. The older population are mainly ***English speaking***
An ***urban area*** with an older population and high levels of ***social and economic*** ***deprivation***. ***Multi-cultural area*** with ***numerous languages*** spoken. We use translators for non-English speaking patients and includes ***refugees*** who are seeking asylum in the UK
Enrolment/registration and consent processes
We only have 45 min for the whole assessment appointment in our service. If we are seeing patients for a first assessment plan to give them the TIPTOE website to have a look at and register or provide a patient information sheet or a QR code to scan. Patients are ***familiar with QR codes*** and ***most have got some form of smartphone***. Many will have already accessed exercises via QR codes and completed MSK patient reported questionnaires
The digital components (registration, e-consent, data collection) may be difficult. The team has done a lot of work around digital access as we have an older population who ***don’t have access to smartphones or the internet***. Need to have options for both digital and non-digital processes. Some with hearing impairment may need face to face. Patients with early-stage dementia may progress into cognitive decline and may no longer be able to engage but we would want to recruit those patients and could encourage them to have a nominated support person. If we recruit via the Pain Service, the multi-stage enrolment process may be perceived as too overwhelming for those who ***live with persistent pain***. Some may struggle with the unfamiliar online tasks that require both energy and concentration. Another concern is that non-English speaking patients represent a large number of our referral numbers however with the literature all in English it is felt they may not be eligible
Data collection processes
The population attending the ***frailty clinic*** are more likely to ‘drop out’ given the nature of the population and they attend for a single assessment appointment so there is ***no ongoing contact***
Clients would probably ***need support*** with data collection, ***many have carers***. Sending it out to them in the post might be quite challenging for some of them. It would be better to do face to face at baseline and support them
Intervention delivery
We anticipate delivering the intervention face to face, potentially blocking off time in MSK clinic. Telephone calls can be provided, but we are not able to deliver the intervention in participants’ homes (our team members are not community-based, the ***time/distance involved*** might be an hour and a half). Quite a lot of ***Welsh speakers*** that would normally receive physio through the Welsh language but currently we will only be delivering the intervention in English language as the Welsh-speaking practitioner is not involved in the trial. People are generally used to travelling for their healthcare appointments and most will be able to access transport or whatever they need to support them to attend. We will need 6 weeks notice period before we are able to allocate/deliver intervention as appointments are booked 6 weeks in advance and patients will be given a standard 40 min slot unless we are able to allocate an appropriate appointment for the trial

### Phase 2: research teams’ attitudes, skills and confidence in recruiting under-served groups

#### Interviews with recruiting staff

Twelve sites were recruited for the initial phase of recruitment. From these sites, 37 recruiting staff were contacted by email between January and October 2024 to confirm their role in the TIPTOE study (recruiter and/or health practitioner) using names and contact details taken from the TIPTOE study database. Of these 37 staff, 13 agreed to be interviewed; 3 whose role was solely a recruiter, 3 as health practitioners, and 7 as both recruiter and health practitioner. In response to lower than anticipated trial participant recruitment, further sites were added which included 6 GP centres from one area in England and a Community Health Care Trust in England. In view of the increase in diversity of sites, further interviews were necessary to explore the full range of sites and their methods of recruitment. From this 2nd recruitment phase, 10 staff members were contacted by email between October 2024 and June 2025, and 4 agreed to be interviewed. In total, 12 interviews with staff recruiters were carried out via Teams. Participants were predominantly female (*n* = 9, 75%), with two-thirds (*n* = 8, 66%) both recruiting participants and delivering the intervention, and one-third (*n* = 4, 33%) recruiting participants only. They included staff recruiting in a range of health boards/trusts across both England and Wales.

#### Findings from interviews with recruiting staff

##### Preparation for recruiting to TIPTOE

Recruiting staff had a range of roles in health and social care and research and had differing levels of experience of recruiting to trials. Some stated that they had not received any TIPTOE-specific training on how to recruit, and they had needed to explore and try out different methods in order to do so.

##### Experience of recruiting TIPTOE participants

**Fig. 2:**
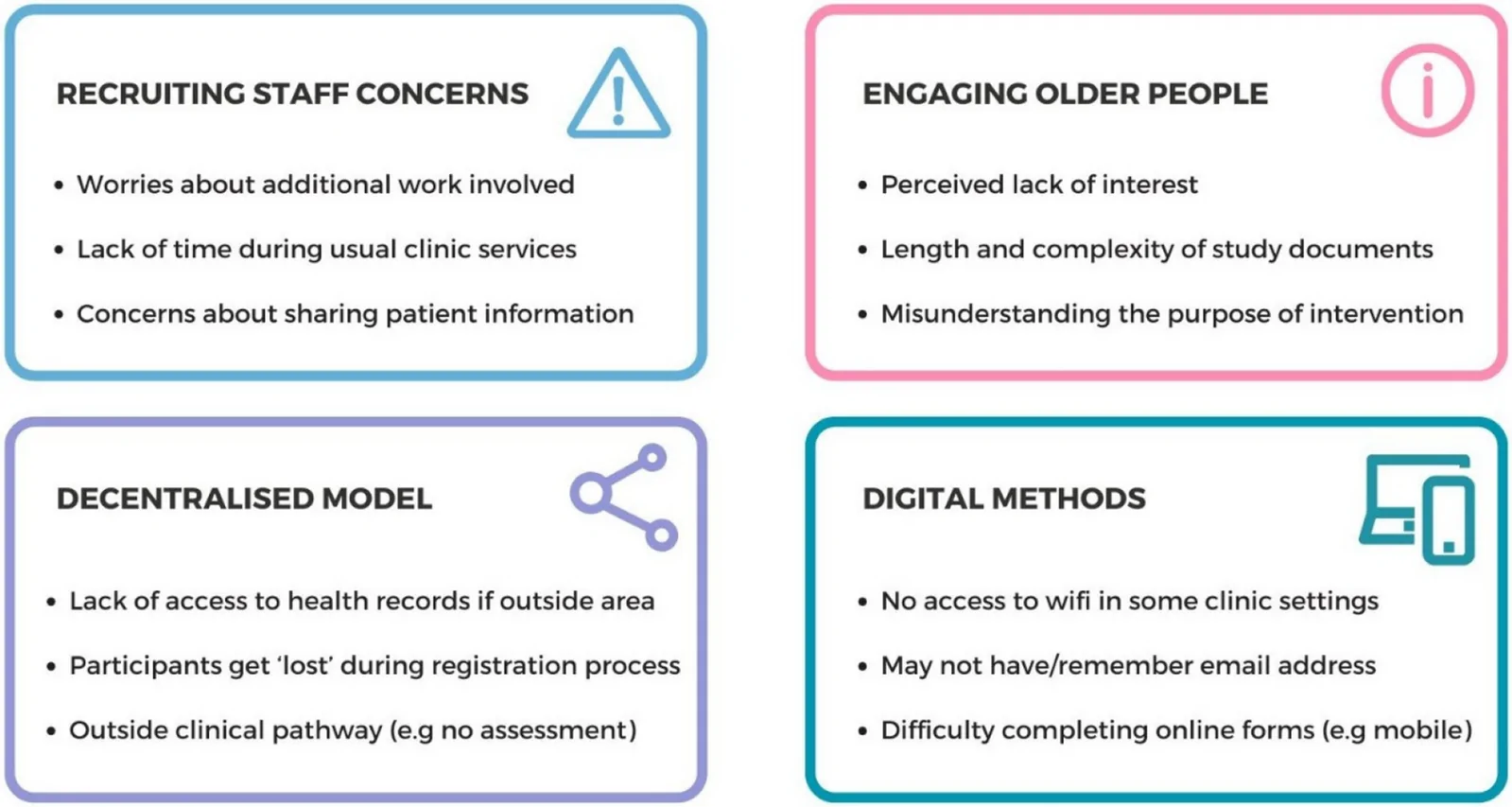
Summary of challenges to recruitment reported by recruiting staff

A range of challenges and facilitators to recruitment were identified by recruiters, which differed by site given the different approaches to recruitment across sites. The challenges are summarised in Fig. 2.

##### Acceptability of the TIPTOE intervention

Some recruiting staff were also healthcare professionals who delivered the TIPTOE intervention whilst others were not at all familiar with it. Therefore, recruiting staffs’ views about recruiting participants to the trial were shaped by their differing levels of knowledge and confidence with the intervention, and consequently their views about the utility of the intervention.

Recruiting staff who were familiar with the intervention reported that the beneficial aspects of using this approach with patients included empowering patients, encouraging more reflection on positive experiences, and offering more time to respond. Some staff reported that only a few participants particularly wanted to be randomised to the intervention arm. Ethical implications of being randomised to the intervention arm of the trial were deemed minimal since safety risk concerns were low in comparison to a drug trial.

However, some staff reported some challenges associated with the intervention. Staff who were not involved in delivering the intervention or were less familiar with a self-management approach were less clear about the ‘added value’ of the intervention beyond the standard care currently available to patients. Staff who did recognise the value of the intervention identified areas, however, where communication to participants about the intervention needed further clarity, particularly where patients’ expectations may differ. For example, participants who were seeking physiotherapy-based or pharmacological interventions may be less accepting of a self-management approach and so may have considered that the trial was less applicable to them.

A lack of participant understanding was also highlighted by some who had expressed an interest in the trial, having no knowledge of the trial and then being contacted by telephone by the recruiter. It was deemed that this could be due to the large volume of trial information for participants, which was sometimes delivered at the same time as attending an osteoarthritis clinic. Some participants did not understand the importance of the usual care arm. Others expected clinical support at their first session when randomised to the intervention arm. Lastly, some proposed that recruitment could be impacted by insufficient resources to deliver the intervention, including time and staff capacities.

##### Potential challenges to inclusivity in TIPTOE

Potential inclusivity challenges that were highlighted by recruiting staff included ethnic minority participants who may experience English language difficulties, those experiencing cognitive decline or learning disabilities, older adults who are hearing impaired, and those with complex health and social problems. Also mentioned were older adults who may be disinclined to participate due to less digital literacy or the lack of an email address. It was suggested that this population may dislike technology and be put off when they hear terminology such as websites, QR codes, and video calls. Furthermore, it was proposed that some patients are not as motivated as others and may be less likely to read trial information, including information about how to participate.

It was also suggested that gatekeeping bias may affect inclusion. For example, those screening patient lists and records may be more likely to exclude older people who they consider would find it difficult to converse or who may be less inclined to manage their own health. Furthermore, it was proposed that recruitment may be directed towards those who are already ‘living well’ with their condition, people who are happy to talk and discuss health issues, and those who could travel to meeting points.

### Phase 3: participants’ experiences of taking part in the trial

#### Interviews with trial participants

In total, 25 older adults with MLTCs participated in an interview during the pilot phase of the trial and the transition to the main trial. Of these, 20 participated in a combined process evaluation and SWAP interview and 5 participated in a SWAP interview only. Participants were predominantly female (*n* = 19, 76%) and had been randomised to the intervention arm (*n* = 20, 80%). In terms of intersectionality with other characteristics associated with being under-served by research, the sample included people from more deprived geographical areas, a range of ethnic backgrounds, those living with frailty, disability (e.g. visual impairment), lower digital literacy, and older age (> 80 years).

#### Findings from interviews with trial participants

##### Receiving initial information about TIPTOE

TIPTOE participants reported that they had been made aware of the trial through a range of routes. These included at a physiotherapy clinic appointment, receiving a letter from the hospital, having prior involvement in a lay university research group or via the ‘Be Part of Research’ registry. Some had received information via social media such as their GP practice highlighting the information on Facebook. Other routes included an information board at an NHS run community exercise programme or from a friend who gave them the TIPTOE trial telephone number to ring.

Participants sampled from under-served groups additionally reported having learnt about the trial through their GP practice either emailing them, sending them a letter, or texting them. However, some could not remember how they had heard about the trial.

##### Motivation for participating in TIPTOE

Some TIPTOE participants reported that they had participated in the trial because they believed the study might benefit themselves or others living with joint pain, particularly because they expressed the view that there is little help currently for older adults living with osteoarthritis. Some participants reported having previously participated in trials, and others that they participated in TIPTOE as they had spare time available or ‘nothing else to do’. This was reinforced by the belief that the TIPTOE intervention was relatively ‘harmless’, did not entail drugs or injections, and that it is an important study to conduct.

Additional reasons for participation stated by those from under-served groups were that the invitation came from their GP, as well as the hope that if they participated they would receive help to explore or manage their joint pain. Some said family members had encouraged them to participate or reported that they had been influenced by having a family member who had previously been involved in research.

##### Views about randomisation in TIPTOE

Participants reported differing views about being randomised as part of the TIPTOE trial. Some expressed a preference for being randomised to the intervention arm, whilst others did not have a preference about which arm of the trial they were randomised to. However, it was apparent that the concept of randomisation was not well understood with some participants believing that the researchers would know best which arm they should be randomised to. Furthermore, some participants stated that they did not understand the randomisation procedure, and some could not recall which arm of the trial they had been randomised to.

##### Facilitators to participating in TIPTOE

Both groups of participants (those in under-served groups and those who were not) expressed the view that participation in TIPTOE was relatively easy. Facilitating factors included that the participant information they were provided with was well-presented, contained sufficient level of detail, and was easy to understand. They had appreciated being able to speak to someone with a ‘good telephone manner’. Indeed, some preferred to engage with the trial using the telephone option, particularly when completing forms. However, others reported that completing forms online was relatively easy. Additional facilitators included having a family member helping them to complete the online forms and also having a medically qualified family member to talk to about the trial. Once again, it was proposed that communication from a GP is beneficial, especially as some expressed concerns about being ‘scammed’ and thought that a telephone call from their GP practice would help reassure them that the trial is genuine.

This was echoed by TIPTOE participants from under-served groups who suggested that being able to participate via the telephone facilitated older adults’ ability to participate as did the friendliness and support of staff. Moreover, it was stated that if the invitation came from a doctor, then participation was more likely. Additionally, one participant who was registered blind reported that the software package that they used to assist magnification worked well with the TIPTOE website.

##### Barriers to participating in TIPTOE

Barriers reported by TIPTOE participants included a concern that their condition was not ‘serious enough’ or their symptoms were not yet challenging enough to warrant trial participation. Some highlighted that the use of a computer could be challenging for older adults who may have only ‘basic computer skills’ and there were concerns about the potential over-reliance or emphasis on computers. Others proposed that the amount of trial information could be perceived as overwhelming and therefore may dissuade potential participants. Moreover, some participants were unaware how the trial would proceed post-randomisation if they were allocated to the intervention arm. Others commented on older adults possibly fearing scams when being contacted, particularly as their friends had not heard about the trial.

Participants from under-served groups highlighted several additional barriers to participation in the trial. Some reported that they felt ‘too old’ to engage in a trial, suggesting that younger people would be more suitable. Some were concerned that if a hospital visit was required, it would have been difficult to arrange the required transport. Some participants cited communication as a potential barrier, including one who reported not receiving a response when ringing the assistance helpline and another stating that they did not understand what the trial was about, even after discussing it on the telephone with a staff member. Others could not remember what the trial was about and claimed that they had not received an information sheet. Another purported that they had a more serious illness which required hospital visits, and so trial participation was deemed comparatively unimportant to them. Some claimed that they would not have participated if the use of a computer was necessary while others stated that it was fairly easy to populate the online forms, but they were described as lengthy. Another stated that the booklet provided as part of the intervention seemed repetitive.

##### Enhancing recruitment to TIPTOE

Participants recommended more publicity be sought for TIPTOE to help raise awareness, such as information being made available through the websites of arthritis charities. They highlighted the vast amounts of leaflets on display in GP surgeries and suggested that leaflets about the trial would be better placed away from these other leaflets and could potentially be given out with appointments. Participants also suggested that there should be greater awareness about TIPTOE amongst their physiotherapy colleagues and community health group leaders, as some had been unaware of TIPTOE. Invitations to participate could also be sent from physiotherapy departments or GPs, as these are seen as reputable sources of information in comparison to a telephone call, which may be deemed a scam. It was also suggested that participants could also be asked to inform their friends as they were likely to be also eligible for the trial.

Participants from under-served groups also recommended GP surgeries participating in the recruitment drive, using telephone and email as the best method to contact potential participants, with less reliance on the use of computers which may discourage older people. However, the use of the telephone was not favoured by all as it was noted that some people may be deterred if they felt they needed to make an instant decision and would prefer to take their time to consider the information before responding, and others may not answer the telephone if the number is unrecognisable. 

## Discussion

The embedded SWAP generated a much greater understanding about the barriers and enablers to including older people with OA and MLTCs in the TIPTOE trial evaluating a self-management intervention, with a particular focus on intersectionality with other characteristics associated with being under-served by research. For TIPTOE, we identified relevant groups as being those in the oldest age categories, linguistic and ethnic minority groups, digitally excluded populations, those experiencing socio-economic disadvantage, and those living with cognitive impairment or frailty or with higher care needs. The findings build on and contribute to a growing body of methodological research on improving inclusion of under-served groups, in response to calls for greater diversity and inclusion in trials [6, 27] including DCTs [28]. It also contributes to the growing demand for better inclusion of older people in research in order to ensure research is more representative of the population who have the highest health and care needs [5, 14, 20] including those with cognitive impairment [29]. It also enabled the TIPTOE trial to proactively address the barriers to inclusion through adapting aspects of the trial in real-time where possible and developing tailored approaches to support the recruitment of these groups.

The site mapping activity demonstrated the heterogeneity of research sites recruiting to TIPTOE and the diversity of populations captured in terms of geography (urban, rural, coastal), ethnicity, language, socioeconomic status, and anticipated levels of health and digital literacy. It also helped to prospectively identify potential challenges to recruitment. This included service-level barriers such as limited appointment times and the notice period needed to pre-book appointments, as well as individual-level issues such as the need for support with data collection, or where delivery of the intervention may be affected by language or other accessibility requirements. It also proved to be a useful discussion and reflection activity with site teams who had not previously considered many of the questions raised and attempted to work through potential solutions during the meetings and beyond.

Interviews with recruiting staff enabled a greater understanding of the barriers and facilitators to inclusive recruitment, including their experiences of approaching potential participants and the reasons given for declining. This was further enhanced with the experiences of older people who had participated in the TIPTOE trial, including those sampled from the under-served groups of interest that we prospectively identified from the literature and across the diverse range of recruitment routes into the study (e.g. via the Be Part of Research registry, frailty clinics, GP practices). This enabled an understanding about areas such as the motivation for participating, issues around digital accessibility and the need for skilled person-centred support, and how a lack of confidence and experience of research can affect both those recruiting and those invited to participate. This led to identifying barriers to include older people with MLTCs in a digitally enabled/DCT, such as how the lack of a direct personal relationship with a clinical or research team may affect older peoples’ trust and commitment to the trial. This included context and population-specific issues such as concerns about being ‘scammed’ when receiving messages from new or unexpected sources, and the challenges of conducting a trial in heterogenous community settings even when the trial is pragmatic, low-risk, and designed with an inclusivity focus. It also provided a greater understanding about the enablers to inclusion, and for recruitment in general, such as maximising flexibility, targeting resources and information, and drawing on wider sources of support such as family members and friends, and other members of multidisciplinary teams.

Concurrent data collection and a rapid analysis approach [24] enabled initial key recommendations to be developed during the pilot phase and the transition to the main phase of the trial. These were used to develop targeted strategies and actions to support inclusion and recruitment more generally. They were also incorporated into the interim report to the funder to provide context to the accruals numbers being reported, specific actions being taken by the trial to address the slower than anticipated recruitment rates, and to justify where and how resources were being prioritised to support inclusion and recruitment. The final recommendations to the TIPTOE trial team are summarised in Table 2.

**Table 2:** Summary of recommendations and actions to improve recruitment and inclusion in TIPTOE

Recommendations to improve inclusion in TIPTOE		Examples of actions implemented during TIPTOE
**Raising awareness about TIPTOE and getting the messaging right**	It was reported that posters and leaflets about the study had limited engagement as there is a lot of competing information. Participants reported that a lot of information was provided about the trial, and this may put people off as it could take time to absorb and could be overwhelming	Recruitment materials were revisited to see if the information could be made more accessible and to highlight key messages (e.g. the study is open to all those 65+ years who are eligible, participation is not just online as there are multiple options, participation in the study is in addition to receiving standard care)
	Participants and recruiting staff suggested exploring other recruitment avenues that may be helpful such as social media which could be aimed at patients and/or their families. Staff suggested considering the use of alternative images to supplement those contained in the TIPTOE book (e.g. additional photographs of older people) and encouraging participants to share their experience with others	In addition to opening additional research site, opportunities to share the information were revisited, including ‘Be Part of Research’ sending invitations at regular intervals (e.g. every 3 months) to new cohorts, and encouraging GP practices to send letters or text messages as appropriate Alongside producing professional social media content, an interview was conducted with a member of the Lay Advisory Group and shared to help highlight the trial to members of the public ‘Taking control and living life to the fullest’—the TIPTOE study supporting people living with osteoarthritis | Health Care Research Wales
	Some staff who were unfamiliar with the intervention were less clear about the ‘added value’ of the intervention beyond the usual care currently available to patients. It was suggested that more guidance would be helpful (particularly for staff who are less familiar with it) about how to describe the self-management approach and its value when approaching potential participants, and to highlight key messages that the intervention is in addition to standard care	A video was recorded for site staff to help them explain the core concepts underpinning the TIPTOE intervention
**Identifying and sharing effective TIPTOE recruitment strategies**	There were reports from staff about the lack of time and resources to recruit participants	Additional central support was provided to aid the recruitment process. However, this had wider resourcing implications for the trial team
	Sites reported that they would welcome more regular communication with the research team about recruitment. They suggested that more support or training for recruitment would be helpful, in addition to learning what other sites were doing to support recruitment efforts. This could be provided through additional opportunities for communicating with the trial team and each other, including sharing practical suggestions between sites	Regular ‘drop-in’ sessions were held with sites to facilitate experience and idea sharing for improved engagement. The central trial team encouraged communication and feedback from sites through opportunities to meet with the trial team on a regular basis, and by collating ‘top tips’ from sites to share via the newsletter. Examples included creating localised forms for clinicians to make it easier to fill in patients’ contact details and medical histories so that the information was to hand before telephoning participants which saved time Options for improving recruitment numbers were explored with sites depending on their context (e.g those who use waiting lists, patient lists and/or those who contact participants in person). Screening case lists were generally seen as more effective than waiting lists or other generic patient lists as patients are already receiving standard care. Suggestions from staff included asking physio students to help with recruitment and using recruitment champions. As First Contact Practitioners (FCP) clinics are highly pressured and short sessions, rather than asking them to constantly recruit it may be more helpful to ask them to do focused periods (e.g. 1 month) of recruitment instead
**Supporting inclusivity in TIPTOE**	It was suggested that amending the eligibility criteria (which was 70+ years at the time) may help widen the opportunity to participate	The inclusion criteria were lowered from 70+ years of age to 65+ years
	Having the option of participants being able to contact someone by phone was highly valued and was seen as key by many who described this population as often not having or using a computer. It was also important to stress to potential participants that they do not need to go online to participate. Face to face sessions were viewed as better than via the telephone or Teams as the former establishes a better rapport and enables participants to ask more questions which led to a better understanding. Recruitment processes need to take account of reports that older people may be worried about possible scams when receiving an invitation to take part in TIPTOE	Where appropriate, sites were encouraged to offer a range of flexible options for participating such as face-to-face options Sites were encouraged to offer the option for participants to have a support person to assist them during the registration and consent process, with questionnaires and data collection, and/or supporting intervention delivery. Questionnaires were reviewed in order to streamline the eligibility and consent forms and the baseline questions as they could involve several communications with the participant including the initial telephone call. For example, the PFFS questionnaire was made non-mandatory before randomisation User rights on the study website/database were adjusted to allow site staff to complete baseline measures with participants in person
	Further guidance may be needed to address gatekeeping and a lack of confidence when recruiting people who may have additional communication and/or capacity needs. Ensure that software required by similar groups of participants is made available, for example people with hearing impairments and those with dyslexia	Guidance was developed for recruiting staff about discussing issues around capacity and consultee involvement in the event of loss of capacity with potential participants (see Appendix)

Recruitment to TIPTOE opened in January 2024 and is ongoing. The intended sample size is 824 participants. When considered against the SEAR (Screened, Eligible, Approached, Randomised) framework which aims to improve the process of recruitment to randomised controlled trials, as of 5th November 2025, a total of 896 people have completed an expression of interest form (EOI), 672 participants have provided consent, and 563 participants have been randomised. This means that even with the benefit of embedding the SWAP, 62.72% of those who completed an online EOI went on to be randomised. Whilst the reasons for the loss of participants during the registration process are not known, it may be due to issues such as a lack of understanding when completing an EOI, or it may be reflective of the challenges around older peoples’ engagement with digital research methods that have been previously reported [10, 14].

The importance of a person-centred approach was highlighted in the SWAP findings; however, there are significant resource implications for providing effective telephone support (e.g. by research nurses with experience of trials involving older people) from the central trial team. The team contacted participants up to three times either by email or telephone, depending on the preferred choice of the participant. A log was kept of telephone calls with participants (to support consent, baseline data collection, or follow up data collection). Whilst some were very brief, for example to arrange a date/time with the participant if they required support, calls to complete baseline questionnaires for example could take up to 45–50 min. Current data shows that of the 672 recruited participants, 357 in total required telephone help at Baseline, 6 month follow up and 12 month follow up (53.1%). Of the 357, the team has successfully helped 277 to date (77.6% of participants). As a breakdown, the number of participants who required help with completing baseline assessments is 138 (38.7%, 117 completed, 21 pending), 6 month follow up is 141 (39.5%, 109 completed, 32 pending), and the number who require help with the 12 month follow up completion is 78 (21.8%, 51 completed, 27 pending help).

### Recommendations for future trials involving older people with MLTCs

Building on the findings from the TIPTOE SWAP and existing guidance for improving inclusion in trials (e.g. [6]) and including older people in research (e.g. [14]), we have developed methodological recommendations to support those designing and conducting future trials (including digitally enabled and/or DCTs) involving older people with MLTCs (Table 3).

**Table 3:** Methodological recommendations to improve inclusion of older people (including digitally enabled and/or decentralised trials)

**1. Ensure a person-centred approach**
When conducting decentralised trials, opportunities should be created to develop and maintain human connections between members of the research team and participants. This could include having a named contact for participants, providing telephone support by staff who have experience with communicating with older people, and leveraging existing relationships such as with the participant’s usual healthcare provider. This is likely to require additional resources and times that should be built into the funding application wherever possible
**2. Get the messaging right**
Participant-facing materials (including websites and non-digital options) should be designed to be accessible, balanced, and convey key information such as the purpose of the trial and intervention, how it compares with usual care, and the participation options (e.g. online, telephone support, in person). Training should be provided for staff who are involved with approaching and recruiting potential participants to help build their confidence and skills with older people with MLTCs. Diverse public involvement contributors should be involved in co-producing participant-facing materials and advising on communication and engagement strategies to ensure that the right message is being conveyed in the best way for this population
**3. Consider intersectionality and digital positionality**
Older people living with multiple long-term conditions may also experience other intersectional factors that can impact their ability and willingness to engage with the study, such as additional communication and/or decision-making needs or socio-economic disadvantage. When using digital methods in a trial, consider how to maximise inclusivity beyond just whether participants have digital access and skills. For example, it is important to seek opportunities to build trustworthiness into the trial design so that potential participants are able to trust the research team, communications about the study, and in the digital technology itself. This could include text messages being sent by the person’s GP or other healthcare provider (rather than from an unknown source), and creating opportunities to build human connections (see recommendations 1 and 2)
**4. Provide flexible support options**
Older people will have individual support needs that should be accommodated where possible, therefore flexible options for participating should be built into a trial. Options could include nominating a support person to assist with completing forms, providing consent verbally (which can be witnessed) or through non-digital means where preferred, ensuring websites and documents are compatible with assistive technology, considering alternative options for sending reminders (e.g. to complete questionnaires) if using auto-generated emails at follow-up timepoints
**5. Avoid losing people through the ‘cracks’**
Where there is a multi-stage process for recruiting participants (e.g. expression of interest, eligibility check, consent, baseline data, randomisation, intervention delivery) consider how best to streamline the process and minimise any opportunities for participants to be ‘lost’ between the stages of the process. Careful tracking of participant movements through these stages, with appropriate actions such as email or telephone reminders, could help reduce the risk of disengagement and withdrawal from the process. This might be particularly the case for participants who are randomised to usual care and hence may receive less contact than those receiving the intervention. This tracking will require additional resource (e.g. data management, trial management) which should be built into the funding application wherever possible

#### Strengths and limitations

Using a mixed-methods approach enabled a broad range of perspectives, including staff involved in recruiting participants and delivering the intervention and older people who had participated in the trial including those from particularly under-served groups. It also captured issues identified prospectively and those that arose in ‘real-time’. Sampling for diversity and across the range of recruitment routes (e.g. via the Be Part of Research registry, frailty clinics, GP practices) enabled an understanding of the common barriers and enablers and those that were context-specific. Using a rapid analysis approach enabled changes to be made to trial processes during the early stages of the trial. It also facilitates future analysis of the process evaluation data as the qualitative researchers are already familiar with the interview data as a result of the SWAP analysis. Combining the interviews for the SWAP and for the process evaluation also provided greater efficiency and could be a model for future studies where inclusivity issues could be incorporated into embedded studies such as process evaluations.

There are a number of limitations to recognise. Interviews were conducted with recruiting staff and participants in TIPTOE and so would not reflect the wider experiences of staff and participants who declined to participate in TIPTOE or those who were not interviewed. The interviews were conducted by phone or Teams and in English, and so those less able or willing to participate in these circumstances were not included. This reflects limitations in the delivery of the TIPTOE intervention as, despite a high level of engagement with under-served populations during the development of the intervention, this is only available in English (or Welsh if there is a Welsh-speaking member of the research team available). This highlights the need for future work to adapt the TIPTOE intervention, should it be found to be effective, to ensure it meets the linguistic, cultural, and social needs of populations with non-dominant language preferences [30].

The SWAP was intended to explore inclusivity issues in TIPTOE as well as other future trials involving older people with MLTCs; however, the findings may not be generalisable to all other trial contexts, for example, where the trial involves a medicinal product, or all populations of older people, for example, those with different accessibility and support needs. As recruitment to TIPTOE is ongoing, we are not currently able to report the impact of the SWAP recommendations on actual recruitment rates or the proportion of those from under-served groups. The findings of the TIPTOE trial, including participant characteristics, will be fully reported once recruitment and data collection and analysis are completed.

## Conclusion

Embedding a methodological ‘SWAP’ study in the TIPTOE trial enabled a greater understanding about the barriers to recruitment faced by older people living with MLTCs, who are often excluded from trials, and in particular those who are from groups who are additionally under-served by research who experience intersectional barriers to inclusion. This included those from the oldest age groups, minority ethnic groups, digitally excluded, and those living with frailty and other conditions requiring higher levels of care and support.

We were able to formulate recommendations for the trial team to support them to rapidly address the barriers through the development of tailored approaches to support the recruitment and follow-up of these groups. This included the need for greater concentration of resources towards providing centralised telephone support for participants who may be less confident navigating online consent and data collection processes, targeted information to raise awareness about the trial through trusted sources such as public contributors, more flexibility when supporting participants to register for the trial and for delivery of the intervention, and the creation of guidance for sites such as around discussing capacity.

In addition to supporting TIPTOE to be more representative of the clinical population, the SWAP findings can support future trials of complex interventions to better include older people with MLTCs and other under-served groups, thus making a useful contribution to the growing body of evidence around recruitment of these populations more broadly.

## Supplementary Information

Additional file 1: Appendix.

Additional file 2: Supplementary file.

## Data Availability

Access to anonymised data may be granted following review of a data request. All data requests should be submitted to: (https://www.cardiff.ac.uk/centre-for-trials-research/collaborate-with-us/data-requests).

## Abbreviations

CTR: Centre for Trials Research, Cardiff University
DCT: Decentralised trial
MLTCs: Multiple long-term conditions
NIHR: National Institute for Health and Care Research
RCT: Randomised controlled trial
SIV: Site initiation visit
SWAP: Study Within a Project
TIPTOE: MulTI-domain Self-management in Older People wiTh OstEoarthritis and Multi-Morbidities
