# Supplementary material for: Addressing the challenges of including older people with osteoarthritis and multi-morbidity in digitally enabled trials: a mixed methods Study Within a Project (TIPTOE SWAP)
Source: Trials. 2026 Jul 27;27:620. doi: 10.1186/s13063-026-09907-w (PMC13685351; doi:10.1186/s13063-026-09907-w)
Supplement: Supplementary file 1 [file 13063_2026_9907_MOESM1_ESM.pdf]

Cardiff University Centre for Trials Research / Prifysgol Caerdydd Canolfan Ymchwil Treialon

FUNDED BY NIHR | National Institute for Health and Care Research

TIPTOE

Guidance for discussing future loss of capacity with TIPTOE participants

Background

As participants are followed up for 12 months in TIPTOE, it is possible that participants who provided consent to take part at the start of the study are not able to continue to consent to their data being collected. The consent they gave at the outset for this type of study is not considered legally valid if they lose capacity.

If these participants are not included (or continue to be included) in the study, it will not provide evidence of the longer-term effect of the TIPTOE intervention for people living with osteoarthritis and conditions such as dementia.
Therefore, an alternative process is needed which involves approaching someone who knows the participant well, such as a family member, to advise about whether the participant would want to continue in the study. This role is called a 'consultee'.

When giving patients information about TIPTOE, we let them know that if for any reason they should no longer be able to consent during the study, we will involve a consultee to advise on their behalf.

The consent form includes a statement about this so that participants can confirm that they understand this is the case. They can also indicate that their wishes are to remain in the study, which can help the consultee when they are asked to advise about what the participant's wishes would be.

Discussions with potential participants

It can be a sensitive topic to discuss with patients, regardless of whether they have a diagnosis of a condition such as dementia. For some patients it may be the first time a topic such as 'mental capacity' has been discussed.

This topic can be introduced into the discussion once the key information about TIPTOE has been covered and questions addressed. It may be best to include this in the discussion about the follow-up period, which is covered in Part B of the main Participant Information Sheet (PIS) after the section about withdrawing for the study.

We have suggested below some ways of approaching the topic when discussing the PIS and consent form.

Possible phrases:

- If any changes happen during this time, for example if you were no longer able to say you are happy to keep being part of the study, we might ask someone who knows you well to advise us about whether you continue in the study.
- This might be whoever acts as your nominated support person, such as <*name of person already discussed, if any*>.
- If <*the person we consult*> advise us that you should continue to be part of the study, we will continue collecting information as usual.
- If they advise that you shouldn't continue, we would withdraw you from the study and no further data would be collected.
- In the consent form you can say what you would want to happen should this be the case at any point.
- This would help <*the person we consult*> to know what your wishes are.

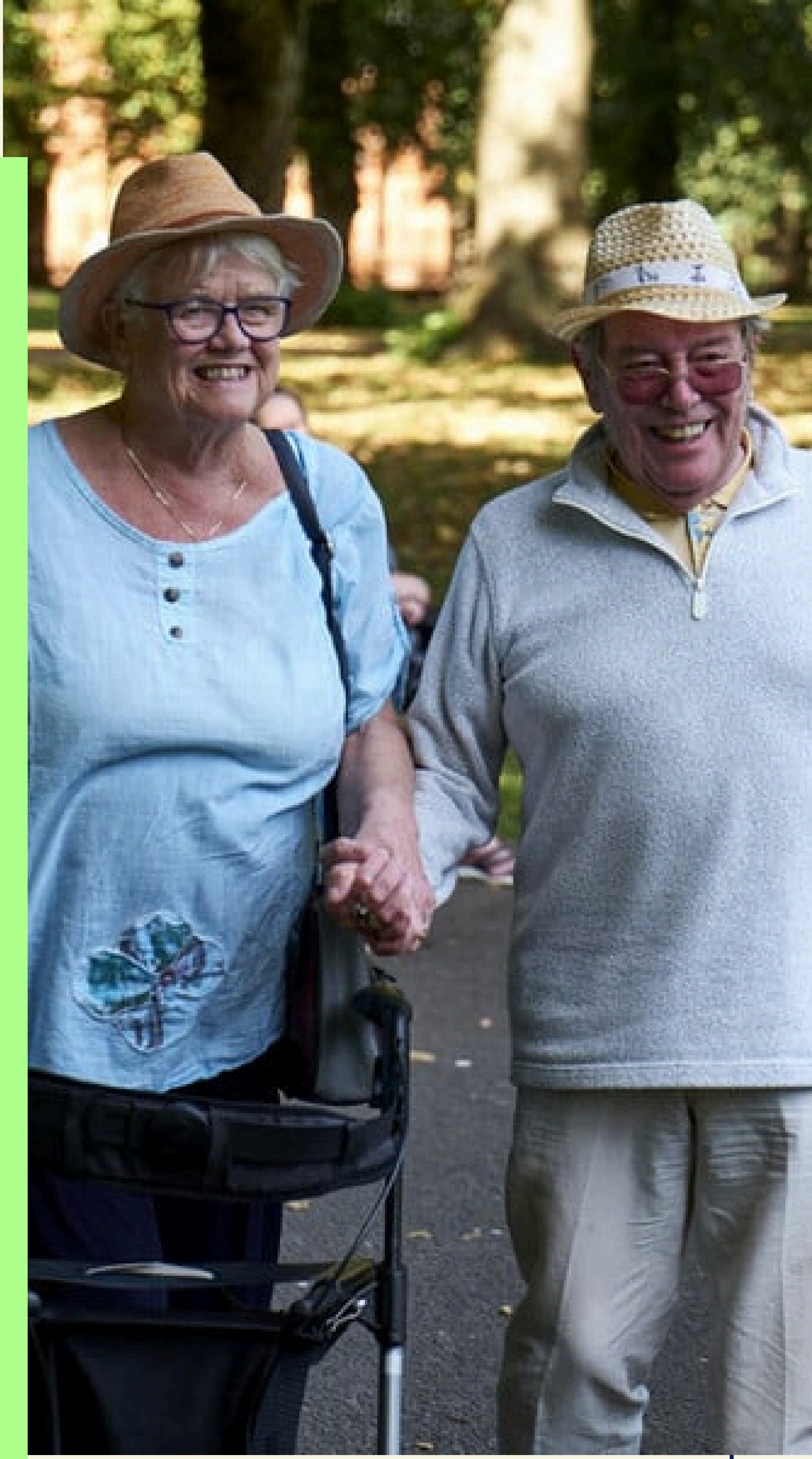

Contact details:

If you have any questions or would like to discuss this, please contact:

✉ TIPTOE@cardiff.ac.uk

MulTI-domain Self-management in Older People wiTh OstEoarthritis and Multi-Morbidities (TIPTOE)
