# Supplementary material for: Addressing the challenges of including older people with osteoarthritis and multi-morbidity in digitally enabled trials: a mixed methods Study Within a Project (TIPTOE SWAP)
Source: Trials. 2026 Jul 27;27:620. doi: 10.1186/s13063-026-09907-w (PMC13685351; doi:10.1186/s13063-026-09907-w)
Supplement: Supplementary file 2 [file 13063_2026_9907_MOESM2_ESM.docx]

**TIPTOE Study Within A Project (SWAP) - Interview Guides**

**Interview guide for TIPTOE Trial Recruiters**

Background

- Can you tell me about your site and your team?
- Do you know what existing joint pain service you have at the site in which you work?
- Do you know how this existing pain service compares to the TIPTOE intervention?
- What motivated you and the site to participate in the clinical trial?

Training

- What were your perceptions of the training?
- Did it cover everything that was required of you in your role?
- Could the training be improved?
- Do you remember the key skills from the Bridges intervention training?

Recruitment

- How did you find recruiting participants into the TIPTOE Trial?
- Do you think that all older adults had an equal opportunity of participating in the trial?
- If some participants did encounter barriers to participation, were these overcome?
- Did you feel that some people would be less suitable for the trial?  If so, did it affect your views about recruiting them?
- Were there any other facilitators or challenges to participating in the trial?
- How was communication with the TIPTOE research team? Did you require any support?
- How could it have been made easier for you to recruit participants?

TIPTOE intervention

- Do you think that the intervention made it easier or more difficult to recruit participants?
- Did you feel that the participants liked or disliked the intervention?

Randomisation

- How did participants feel about being randomised to the intervention or control group?
- What are your thoughts on the equipoise of the trial?

Trial documentation

- What did you think of the participant information sheets and consent forms?
- If you were involved in the consenting process, how was that?

**Interview Guide for TIPTOE Trial participants (including from under-served groups)**

Recruitment

- How did you hear about the TIPTOE Study?
- Did you have easy access to the information?
- What information did you remember most?
- Did you find the study information documents easy to read and to understand?
- How easy was it to decide about taking part in the study?
- Why did you decide to participate?

Participation

- In general, how easy was it to take part in the study e.g. registration, consenting and filling in questionnaires?
- What did you think of the resources associated with the study eg the website and the booklet?
- What was it like to be a participant on the trial?
  - if you were receiving the intervention sessions, did you need help in any way to become involved with these sessions?
- What could have been done to make it easier for you to take part in the study?
